# Longitudinal strain from velocity encoded cardiovascular magnetic resonance: a validation study

**DOI:** 10.1186/1532-429X-15-15

**Published:** 2013-01-23

**Authors:** Einar Heiberg, Ulrika Pahlm-Webb, Shruti Agarwal, Erik Bergvall, Helen Fransson, Katarina Steding-Ehrenborg, Marcus Carlsson, Håkan Arheden

**Affiliations:** 1 Department of Clinical Physiology, Lund University, Lund University Hospital, Lund, Sweden; 2 Centre for Mathematical Science, Lund University, Lund, Sweden

## Abstract

**Background:**

Regional myocardial function is typically evaluated by visual assessment by experienced users, or by methods requiring substantial post processing time. Visual assessment is subjective and not quantitative. Therefore, the purpose of this study is to develop and validate a simple method to derive quantitative measures of regional wall function from velocity encoded Cardiovascular Magnetic Resonance (CMR), and provide associated normal values for longitudinal strain.

**Method:**

Both fast field echo (FFE) and turbo field echo (TFE) velocity encoded CMR images were acquired in three long axis planes in 36 healthy volunteers (13 women, 23 men), age 35±12 years. Strain was also quantified in 10 patients within one week after myocardial infarction. The user manually delineated myocardium in one time frame and strain was calculated as the myocardium was tracked throughout the cardiac cycle using an optimization formulation and mechanical *a priori* assumptions. A phantom experiment was performed to validate the method with optical tracking of deformation as an independent gold standard.

**Results:**

There was an excellent agreement between longitudinal strain measured by optical tracking and longitudinal strain measured with TFE velocity encoding. Difference between the two methods was 0.0025 ± 0.085 (ns). Mean global longitudinal strain in the 36 healthy volunteers was −0.18 ± 0.10 (TFE imaging). Intra-observer variability for all segments was 0.00 ± 0.06. Inter-observer variability was −0.02 ± 0.07 (TFE imaging). The intra-observer variability for radial strain was high limiting the applicability of radial strain. Mean longitudinal strain in patients was significantly lower (−0.15± 0.12) compared to healthy volunteers (p<0.05). Strain (expressed as percentage of normal strain) in infarcted regions was lower compared to remote areas (p<0.01).

**Conclusion:**

In conclusion, we have developed and validated a robust and clinically applicable technique that can quantify longitudinal strain and regional myocardial wall function and present the associated normal values for longitudinal strain.

## Background

Quantitative regional wall motion analysis is important for diagnosis, physiological understanding of the heart, and to evaluate treatment strategies for heart disease. Today regional wall function is typically assessed visually and may suffer from inter-observer variability and require experienced readers. The use of strain as a quantitative measure of regional function has been limited due to a lack of robust analysis techniques.

Quantification of tissue deformation and strain in human myocardium has been undertaken using various techniques such as tissue Doppler echocardiography [1,2], speckle tracking [3], cardiovascular magnetic resonance (CMR) with tagging [4-7], and velocity encoding [8-11] techniques. DENSE (Displacement Encoding with Stimulated Echoes) is a CMR technique where displacement of myocardium is encoded in the phase of the images. It allows calculation of displacement maps that can be directly differentiated into strain maps [12]. Current post processing includes promising approaches for processing of DENSE images including myocardial tracking [13]. Another method of achieving simplified quantification of strain is HARP (HARmonic Phase) where applied tagging modulates the underlying image with spectral peaks in the k-space. The idea is to isolate the first peak that contains the tagging information. This results in both a phase image and a magnitude image that is combined to a HARP image that contains the deformation of the myocardium encoded in the phase [7]. Yet another technique to measure strain from CMR is SENC (Strain ENCoded CMR) that is related to DENSE by the fact that they both utilize stimulated echoes. In contrast to DENSE, SENC is obtained from the magnitude images and incorporates modulation in the through plane direction. Two images are acquired at different tagging frequencies and by combing the information a colour coded strain map can be obtained that allows strain mapping on a pixel by pixel level [7]. One disadvantage that HARP, DENSE and SENC all share is the limitation of low SNR due to pulse sequence design or the processing nature. Tagging techniques such as SPAMM (SPAtial Modulation of Magnetization) or C-SPAMM, DENSE, HARP and SENC have all been used in several research studies. The clinical applicability of the aforementioned techniques is still limited, and task force reports on standardized imaging protocols for clinical CMR does not even mention any of the above techniques for analysis of regional LV function [14].

To our knowledge all previous studies that have attempted strain analysis from velocity encoded imaging used two dimensional short axis images or full three dimensional approaches. In this study we propose the use of long axis images. The advantage of using long axis images is the smaller through plane motion in the imaging plane, which is important in the tracking process. By using long axis images it may be possible to measure longitudinal and radial strain with small assumptions on myocardial motion.

In order to make a robust and stable tracking algorithm, we propose a tracking algorithm which utilizes mechanical *a priori* information in the tracking process, which is lacking in the previously proposed methods.

Previous methods for strain quantification have been around for almost a decade, and still they have found limited applicability in research and in the clinic, besides a relatively small number of selected research studies compared to other CMR techniques. This can be attributed to the fact that many of the previously published methods require tedious manual analysis and that suitable dedicated software for analysis has been unavailable to the research community.

Therefore, the aim of this paper is to provide a quantitative and clinically applicable method for calculating strain, and provide associated normal values for the presented method.

## Methods

### Study population

Healthy volunteers were recruited through advertising. Exclusion criteria included history of heart disease, hypertension, diabetes, and pregnancy. Volunteers underwent a physical examination, resting electrocardiogram (ECG), CMR with velocity encoded imaging, and exercise stress testing. Volunteers with abnormal stress tests and incomplete CMR were excluded. Thirty six volunteers were included in this study (13 women and 23 men, mean age 35 years, range 21–65 years). All volunteers were moderately physically active but none were reported to be an active athlete. None were obese (mean BMI 24.0, range 18–30). All were in sinus rhythm with a mean resting heart rate 61 beats per minute (44–83) and the mean blood pressure was 127/75 (range 110–145/65–90 mmHg). All patients had normal ejection fraction and cardiac dimensions.

To contrast the healthy volunteers, we included 10 patients 1 week after myocardial infarction (2 women and 8 men, mean age 64 years, range 39–85 years).

This study was approved by the regional ethics committee and written informed consent was obtained from all participants.

### CMR

Steady state free precession images in the 2CH, 3CH and 4CH imaging planes were acquired with a 1.5 T Gyroscan Intera Scanner (Philips Medical Systems, Best, the Netherlands). Imaging parameters were TR/TE: 3.4/1.5 ms, flip angle = 60°, image resolution 1.36 × 1.36 mm, slice thickness 8 mm, retrospective ECG gated reconstruction. Short axis image stacks using steady state free precession images were also acquired. Imaging parameters were TR/TE: 2.8/1.4 ms, flip angle=60°, typical image resolution 1.5 × 1.5 mm, slice thickness 8mm, retrospective ECG gated reconstruction. Thereafter, two types of two-dimensional in plane velocity data were acquired; gradient field echo images (also called fast field echo images, FFE) and turbo gradient field echo images (TFE images). The velocity data were acquired in the same image planes as the anatomical images, and the two velocity encoding directions were interleaved in the same heart beat. For the patients, late gadolinium enhanced (LGE) images were acquired approximately 20 minutes after contrast injection of 0.2mmol/kg extracellular gadolinium based contrast agent (gadoteric acid; Guerbet, Gothia Medical AB, Billdal, Sweden). LGE images were acquired with an inversion recovery sequence with field of view 340 mm, flip angle = 25°. TR/TE: 3.14/1.58 ms.

For FFE images typical imaging parameters were TR/TE: 24/4.5 ms. Acquisition was performed during free breathing, typical imaging time was about one minute and no navigator was used. Temporal resolution was typically 16–22 time frames covering the whole cardiac cycle. SENSE was not used. The number of reconstructed time frames was the same as the number of acquired time frames.

For TFE images typical imaging parameters were TR/TE: 24/5.3 ms. Acquisition was performed in end expiratory apnoea and typical imaging time was 15–25 s. TFE echo train length was 5 (echoes per excitation). Temporal resolution was typically 12–16 time frames covering the whole cardiac cycle. The number of reconstructed time frames was the same as the number of acquired time frames.

For both FFE and TFE images flip angle was 15°, velocity encoding was set to 20 cm/s. Saturation bands (30 mm thickness, 30 mm gap to image plane) superior and inferior to the imaging slice were used to reduce blood flow artefacts, which increase signal to noise [15]. Image resolution was typically 1.5 × 1.5 mm, and slice thickness 8 mm. Retrospective ECG gating was used when reconstructing the images. The images were reconstructed to the acquired spatial resolution and no zero-filling was used.

### Phantom experiments

**Figure 1:**
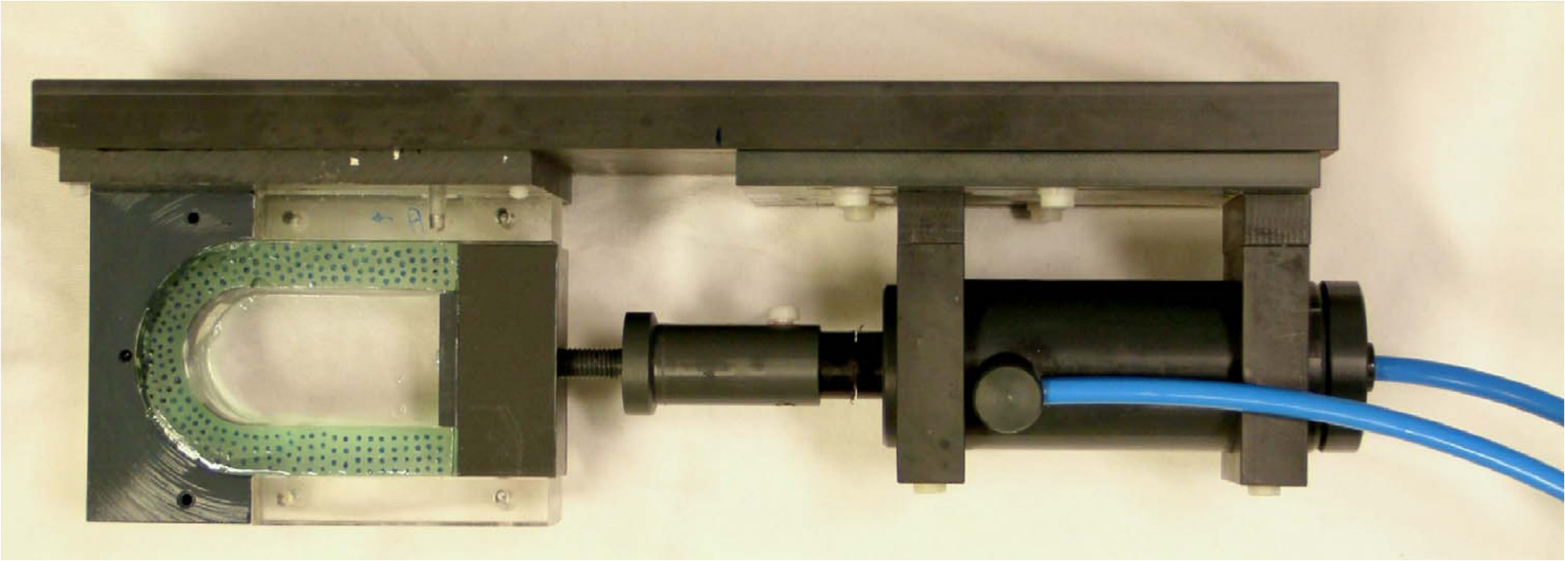
**Phantom experimental setup.** A U shaped gelatine cast is compressed by a pneumatic driven compression device. The gelatine was painted with a grid pattern to allow for optical tracking of the deformation as ground truth.

A U-shaped gelatine phantom measuring 100 × 70 × 15 mm was constructed to simulate a long axis slice of the left ventricle. The phantom was made using 1 tablespoon powdered gelatine first dissolved in 30 ml of cold tap water and then in 100 ml of near-boiling solution with 2 mmol CuSO4. We used CuSO4 to get increased signal and T2 and T1 relaxation times corresponding to normal myocardial tissue. The gelatine mixture was poured into a U-shaped mould and chilled overnight. We designed an MR compatible pneumatic driven compression device (Figure 1). The phantom was longitudinally compressed approximately 15 mm. An animation of the phantom setup is shown in the Additional file 1.

### Delineation of the myocardium

The first step in the strain calculation is manual delineation of the left ventricular myocardium in end diastole of the anatomical steady state free precession cine images. Thereafter, the segmentation is transferred onto the velocity encoded images. The reason for using delineation of the steady state free precession images is the high image contrast compared to velocity encoded images. When required, the delineation was modified on the velocity encoded images.

### Image analysis

The myocardial contour is tracked using a novel tracking approach throughout the cardiac cycle based on the acquired velocity information. Details about the tracking algorithm are given in the Additional file 2.

In short, inside the myocardium, a set of 7 local basis functions are equally distributed. Each basis function controls the local deformation of the myocardial model. The deformation field (coefficients for the local basis functions) is calculated by minimizing the difference between the measured velocity and the deformation using partial differential equation formalism. In order to allow for manual corrections an additional term in the optimization was included (minimizing the distance between node points on the boundary and optionally placed correction points in time and space). A material model was used to calculate the displacement of any interior point based on a deformation of the contour. The Lagrangian strain tensor was calculated from the deformation field and then decomposed into the radial and longitudinal directions. The longitudinal axis was defined as parallel to the centre line of the myocardial delineation. The radial axis was defined as orthogonal to the longitudinal axis. Calculation time is about one second on an ordinary PC. Global strain was calculated as mean strain over the entire myocardium.

### Implementation

The proposed algorithm to calculate strain together with user friendly tools for manual corrections, visualization, normal values and comparison tools are all implemented into the software Segment v1.9 R2394 ( http://segment.heiberg.se) [16]. An illustration of the complete process using the software is illustrated in Additional file 3.

### Manual corrections

Manual corrections were applied if necessary in the end systolic time frame. The contour of the tracked myocardium was overlaid onto the steady state free precession images. Manual corrections were applied if necessary in the steady state free precession images and automatically transferred to the velocity encoded images. For all cine acquisitions, no more than 17 correction points were added.

### Validation experiments

The phantom setup was scanned with the same imaging techniques as the human subjects. In order to validate phantom strain measurements we used optical point tracking as an independent technique. On the surface of the phantom a grid pattern was created with a water proof pen. After CMR acquisition the phantom setup was brought out from the scanner and was immediately filmed with a Canon 7D camera at a frame rate of 50 fps and image resolution 1080 × 720 pixels. The CMR acquisition was performed with the same TFE imaging sequence as the healthy volunteers, except that triggering was modified to accommodate for the longer RR-interval.

A custom software was developed in Matlab ( http://www.mathworks.com) to perform optical tracking of the grid points, and calculate strain from the measured displacement field. Each frame in the movie sequence was processed with a filter that was matched to the size of the grid points. In a first step a user semi-automatically identified grid points. The points were automatically tracked using their position in the previous frame and knowledge of piston movement. After this initial guess all points were moved to the nearest local maxima on the filtered images. This process was repeated for all timeframes. From the tracked position of the grid points, a sparse displacement field could be calculated. This displacement field was up-sampled using cubic interpolation to a dense regular deformation field, which allowed strain tensor calculation by numerical differentiation.

The proposed velocity encoded CMR based strain method was compared to the result of strain quantification using optical tracking. The comparison was made over time in three region of interest.

To test intra-observer variability, one observer (UPW) outlined the myocardium and performed manual corrections of the strain calculations in 10 subjects twice. This process was performed both for FFE and TFE images. The strain measurements were performed about one week apart.

Furthermore, to test inter-observer variability another observer (EH) measured strain on TFE images from 10 healthy volunteers. The myocardial delineation and manual corrections were performed independently. Inter-observer variability was also assessed for the patients by comparing results from two observers (UPW and EH, respectively).

In order to test if the different velocity acquisition techniques (FFE and TFE, respectively) could be used interchangeably one observer measured strain in 10 subjects using both techniques. The same myocardial delineations were used for both the FFE and TFE images to limit the influence of manual corrections.

Strain was measured in 10 patients one week after a myocardial infarction in infarcted regions and compared to strain in remote myocardium of the same patients. Remote myocardium was defined as sectors in the 17 segment models where the mean of transmurality of adjacent sectors did not exceed 25 percent and the sector itself did not contain any infarct. Infarcted regions were defined as having any infarction in the area. Strain was expressed as percentage of the strain in the healthy volunteers by dividing the measured strain with the mean strain from the respective sector in the healthy volunteers.

### Statistical analysis

Inter- and intra-observer variability was calculated by taking the difference between observer one and observer two. For all differences the mean (bias) and standard deviation (variability) was calculated. Comparison between strain using optical tracking and strain using velocity encoded imaging was performed by Bland-Altman analysis. Values are presented as mean ± SD. Correlation between global longitudinal strain and age was done by Pearson coefficient of correlation. Statistics were calculated with Matlab, version 2011a, ( http://www.mathworks.com). Level of statistical significance was set to p<0.05.

## Results

**Figure 2:**
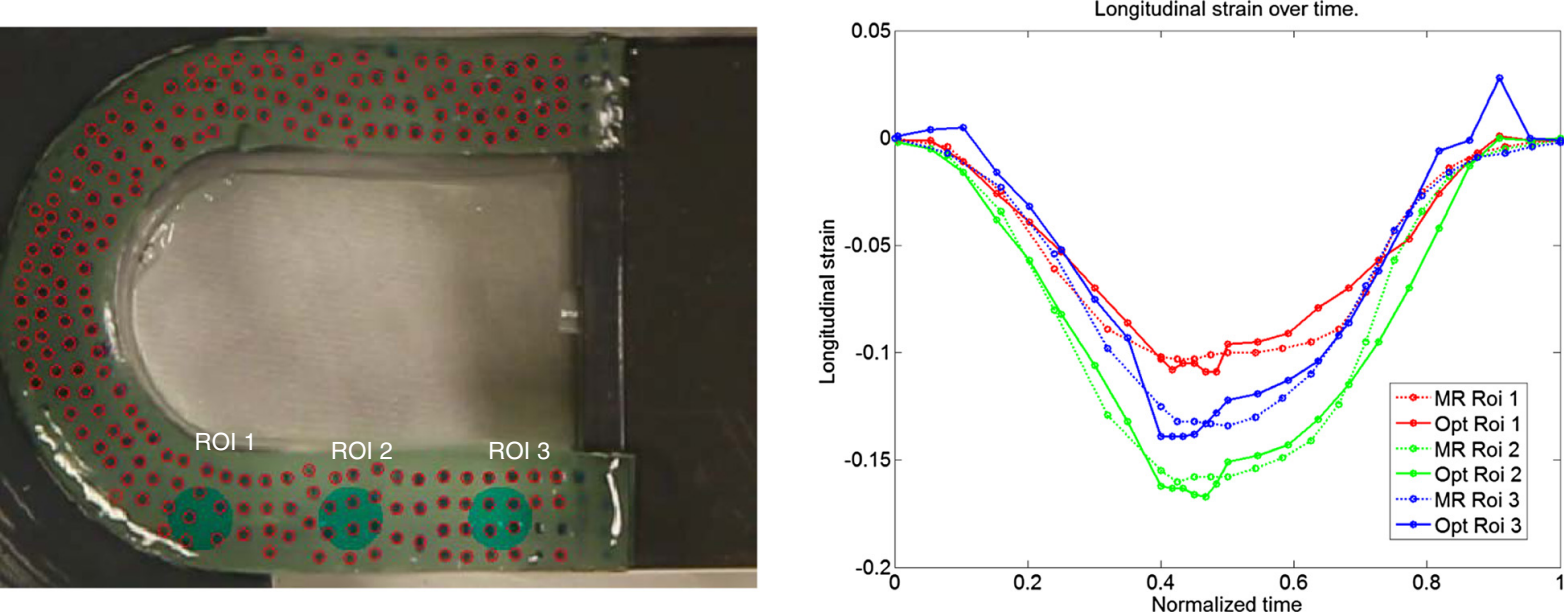
**Phantom experiment.** Left panel shows the placement of the ROI's and the result of the optical tracking as red dots. Right image panel shows longitudinal strain for the three different ROI's (red, green, and blue color) with the optical method (solid lines) as well as the CMR method (dashed lines).

**Figure 3:**
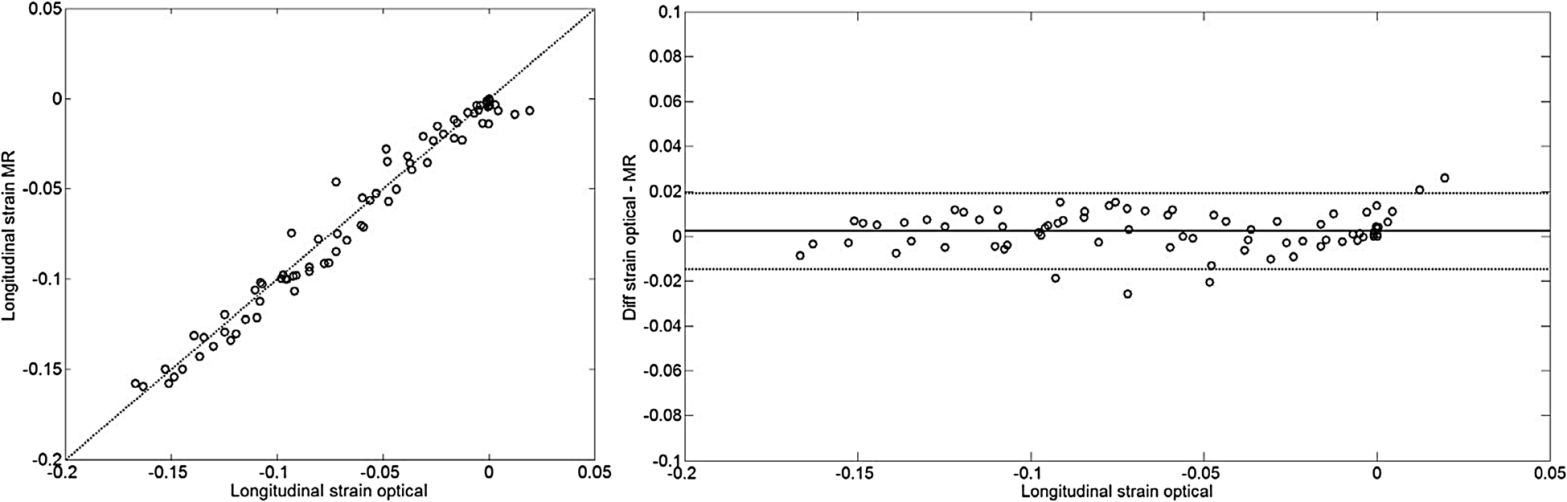
**Result for the three ROI's and all timeframes.** Left panel shows a correlation plot between longitudinal strain using the optical method and longitudinal strain using MR. The dashed line indicates the line of identity. Right panel shows a difference plot, where the difference between the methods is on the y-axis and the longitudinal strain using the optical method on the x-axis. The solid line indicates the mean difference and the dashed lines indicate 2SD.

There was an excellent agreement between CMR tracking using TFE imaging and optical tracking to quantify longitudinal strain over time (Figure 2). An animated version of the result of the optical tracking is shown in Additional file 4. An animated version of the CMR tracking is shown in Additional file 5. The bias between longitudinal strain using optical tracking and CMR was 0.0025 ± 0.085 (Figure 3).

**Figure 4:**
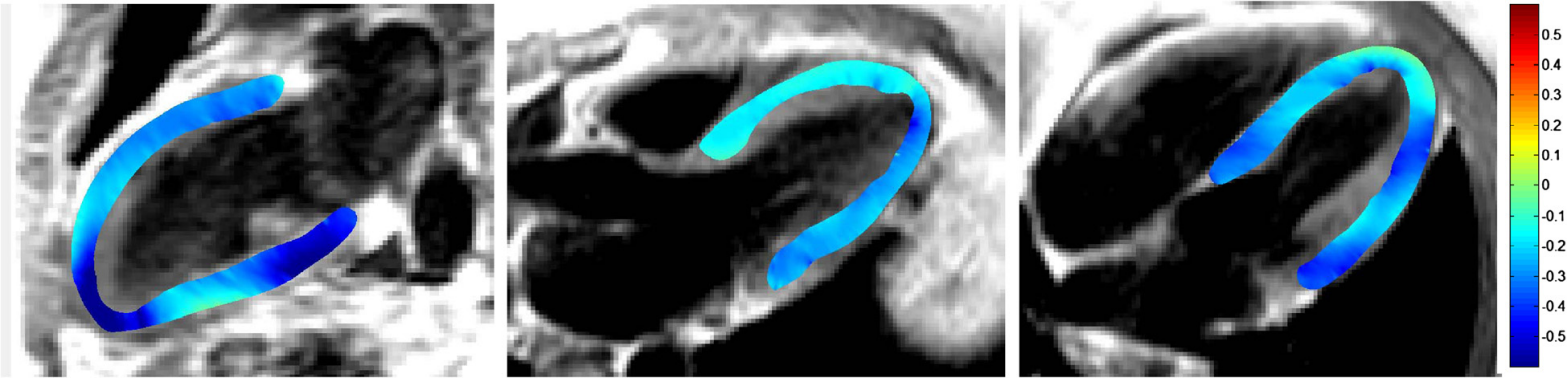
**A typical example of longitudinal strain in systole in a normal volunteer.** Left image panel shows the 2CH view. Middle image panel shows the 3CH view, and the right image panel shows the 4CH view.

**Figure 5:**
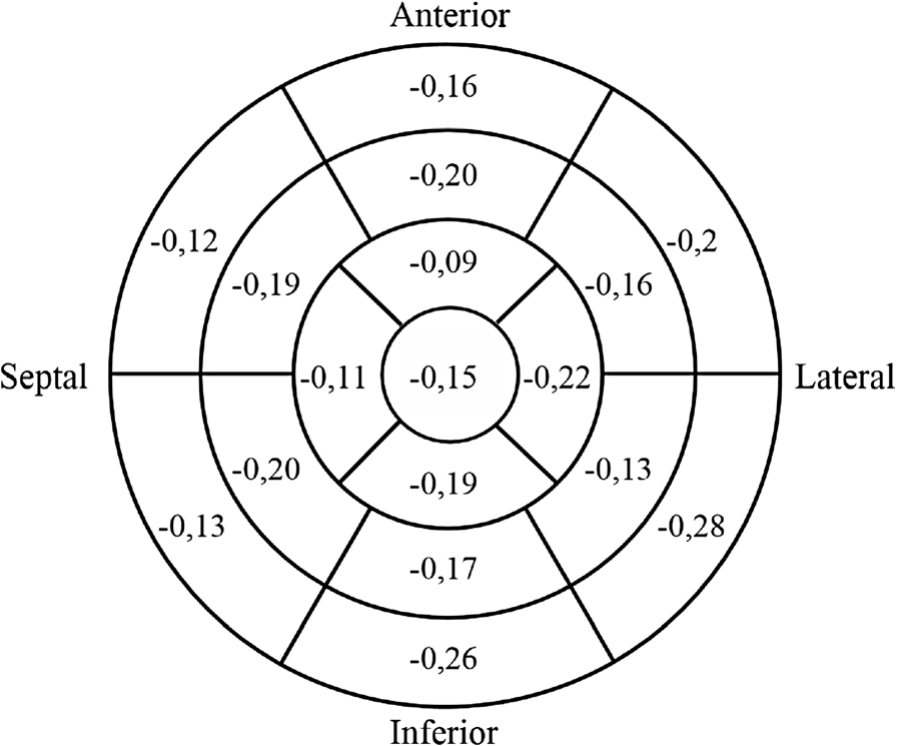
Mean of longitudinal strain from 36 healthy volunteers presented in a 17 segment model.

A typical example of longitudinal strain in systole is shown in Figure 4. An animated version is available in the Additional file 6. Mean global longitudinal strain in the 36 healthy volunteers was −0.18 ± 0.10. Longitudinal strain per sector is shown in Figure 5. There was no correlation between longitudinal global strain and age (r^2^ = 0.097, p=n.s).

Intra-observer variability for all segments on FFE images was −0.01 ± 0.11. Intra-observer variability for mid-ventricular and apical segments on FFE images was 0.00 ± 0.07 and for basal segments −0.03 ± 0.11. Intra-observer variability for all segments on TFE images was 0.00 ± 0.06, for mid-ventricular and apical segments 0.00 ± 0.05, and for basal segments 0.02 ± 0.07. Inter-observer variability on TFE images was −0.02 ± 0.07 for all segments, 0.00 ± 0.06 for mid-ventricular and apical segments, and −0.04 ± 0.08 for basal segments. Inter-observer variability in patients was −0.03± 0.07.

The above analysis was also performed for radial strain using TFE images. We found that the intra-observer variability for radial strain was high (0.10 ± 0.33) limiting the applicability of radial strain in this setting.

**Figure 6:**
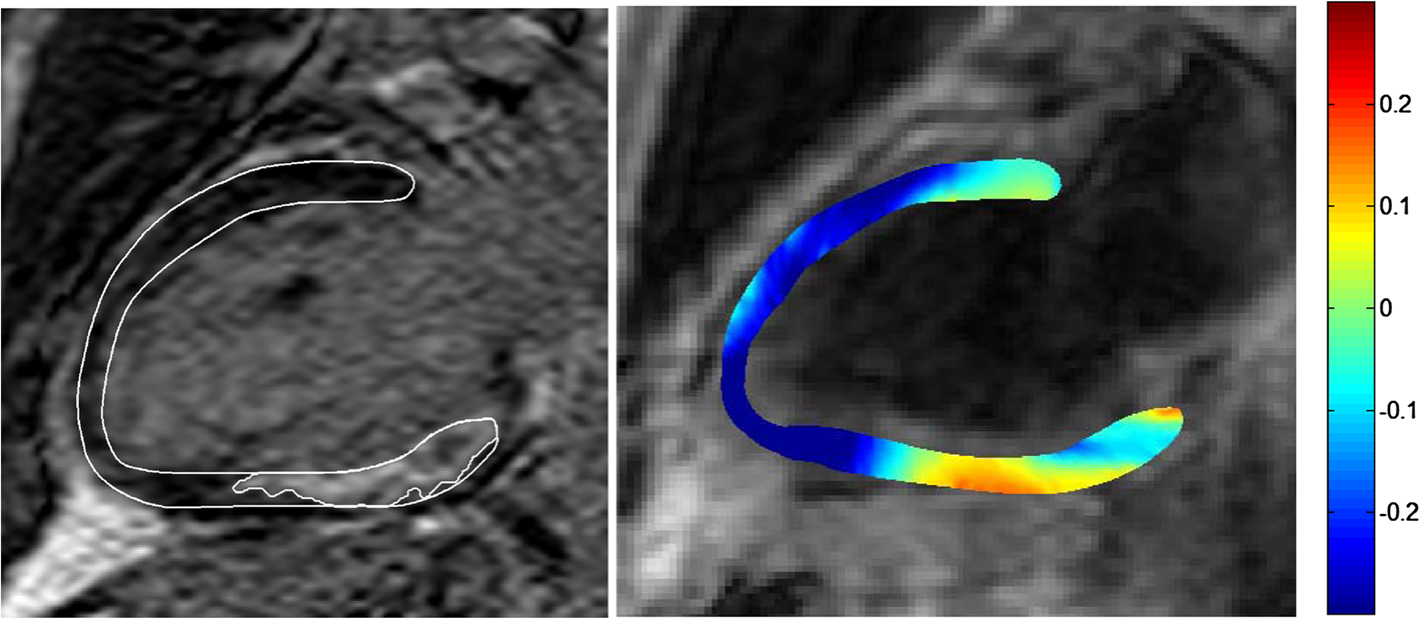
**Two chamber view of strain in a patient with an inferior infarct.** Left panel shows a late gadolinium enhancement image with the infarct region outlined. Right panel shows longitudinal strain where the color overlay indicates strain values.

Mean longitudinal strain in patients was −0.15 ± 0.12 (measured in TFE images). The mean longitudinal strain in the patients was lower compared to healthy volunteers (p<0.05). One patient had an aborted infarction and was excluded from further analysis. The mean number of remote sectors in the patients was 8 segments (range 3–11 segments) out of the total 17 segments. Mean longitudinal strain in the remote regions was 88 ± 27% of the strain in corresponding sectors in the healthy volunteers. Mean longitudinal strain in the infarcted regions was 60% ± 27% of the strain in corresponding sectors in the healthy volunteers. Strain (expressed as a percentage of normal strain) in the infarcted regions was lower compared to remote areas (p<0.01). Figure 6 shows an example with a patient with an inferior infarction, and a corresponding animation is found in Additional file 7.

## Discussion

We have developed and validated a method to quantify longitudinal strain from velocity encoded images. The complete analysis takes in the order of a few minutes, including delineations and manual corrections (Additional file 3). As such, the presented method has potential to quantify strain in a clinical setting.

The results from the phantom validation show that the proposed velocity encoded CMR based strain method can accurately measure longitudinal strain compared to optical tracking as an independent gold standard.

Values for longitudinal strain in the present study compare well with those previously presented by use of myocardial tagging. Comparing with the study by Moore *et al*. [17] on 31 healthy volunteers the longitudinal strain was −0.16 versus −0.18 in this study (measured as mean global longitudinal strain from TFE imaging). It also compares with values from Bogaert et al. who found global longitudinal strain of −0.17 versus −0.18 in this study [18].

Inter- and intra observer variability was comparable (0.00 ± 0.06 versus −0.02 ± 0.07, respectively). Differences were more prominent in the basal regions, where the deformation is slightly higher and the motion is more complex.

Total longitudinal strain was significantly lower in patients one week after a myocardial infarction. Furthermore, we showed that using the proposed method to quantify strain it was possible to differentiate strain in affected regions versus remote regions in 10 patients. The patients were significantly older than the healthy volunteers, and this may have contributed to the difference between the two groups. However, we did not see significant correlation between age and global strain in healthy volunteers.

Inter-observer variability for FFE images was −0.01 ± 0.11 compared to inter-observer variability on TFE that was 0.00 ± 0.06. This is consistent with a visual impression that it was easier and more robust to quantify strain using TFE imaging. One reason for the more challenging analysis on FFE images could be the presence of ghosting artefacts caused by breathing motion since the FFE images were collected during free breathing. Another reason for the lower inter-observer variability can be that the delineations were transferred from steady state free precession images to the velocity images and both TFE and steady state free precession images were acquired during breath hold. Manual corrections of the delineations were performed both for TFE and FFE images when deemed necessary. The fact that inter-observer variability was comparable in both healthy volunteers and in patients and that strain in infarcted regions was lower than in remote myocardium indicates that the *a priori* mechanical assumptions seems to be reasonable and repeatable also in pathological cases.

Although the software calculates radial strain we found that quantifying radial strain in this setting was challenging. We believe that the difficulties are related to voxel size in comparison to typical wall thickening. A typical wall thickening is about 50%, and this translates to a thickening in the order of 2–4 mm. This should be contrasted to the voxel size that is in the order of 1.5 mm.

One advantage with the proposed technique is the simple analysis process, compared to other tagging based methods that can be tedious. Tag fading have improved with recent pulse sequences, but might still cause problems when analysing the end of the cardiac cycle.

An important clinical application is to quantify regional function in both stress and rest conditions. Increased heart rate under stress conditions will increase the mean myocardial tissue velocity, while the amount of global deformation is approximately the same. Strain rate is defined as the difference in velocity between two adjacent points in the myocardium divided by the distance between the two points. Myocardial velocities will increase as systole is shortened during stress conditions. If we assume that the entire myocardium increase contraction velocity uniformly, the difference in velocity between two points in the myocardium will increase as the overall velocity increase. Under this assumption, strain rate will also increase as it is linearly proportional to the velocity differences. Strain is not related to the rate of deformation and only the total deformation, and is therefore likely to be heart rate independent and is thus a better candidate to quantify regional wall function in stress examinations.

To be able to use the presented technique for stress examinations, the temporal resolution needs to be increased by a factor of about 2.5 times. This can be expected to be met in the future by using SENSE in multiple coil settings and the rapid development of new pulse sequences and novel acceleration schemes such as K-T-BLAST [19], or if possible to sacrifice spatial resolution.

To our knowledge this approach is different compared to all previous methods for deriving strain from velocity encoded imaging in that previous methods did not use a material model to track interior points. Another difference compared to previous studies is that to our knowledge all previous studies have attempted strain analysis from velocity encoded imaging using two dimensional short axis images or full three dimensional approaches. One limitation with the proposed method is that it only uses in-plane velocity encoded long axis images. As a result it will invariably ignore any torsion or rotation of the left ventricle and not provide information about the circumferential strain. We chose to use long axis images instead of short axis images, since the torsion of the normal left ventricle is smaller than the long axis motion and the error in neglecting one velocity component will subsequently be smaller. A large degree of rotation might introduce problems in the tracking since through-plane motion will mean that we are not following the same myocardium over time. Rotation is higher in the apex compared to the base and therefore, this might affect the results in the apex. The advantage of long axis images is the smaller through plane movement which is important in the tracking process. Strain from long axis imaging is also commonly used with the SENC method [20]. The ideal approach would be to use full three dimensional volume with all velocity components as used in previous studies [21].

At present the acquisition time for a navigator gated 3D image stack is about 10–15 min, which is not suitable for clinical routine, specifically not for stress strain image acquisition. A combined approach where long axis images are combined with some short axis images would most likely be feasible, and this approach has been taken by several tagging studies [17,18,22,23].

There are other existing techniques to derive strain, such as DENSE, HARP, and SENC. These have the possibility to derive quantitative strain information with processing times comparable or even faster than our proposed method. They have a common weakness in low SNR, and it may be that the post processing algorithms for these techniques lack tools for rapid manual corrections in cases of noise or incorrect myocardial tracking. The strength with the presented method is in the robustness of the tracking and the ease and possibility of manual corrections if necessary.

## Conclusion

In conclusion, we present and validate a robust and clinically applicable technique to quantify longitudinal strain and regional myocardial wall function with associated normal values.

## Competing interests

EH is major share holder of Medviso AB, which produces cardiovascular imaging software. No funding has been received from any non public funding source. No other authors have any competing interests to disclose.

## Authors’ contributions

HA, EH and UPW conceived the study. EH, UPW, and SA, analysed and interpreted data. EH, SA, HF, MC and EB all worked on the practical implementation of the method. UPW, EH and MC performed phantom experiments. KSE included all volunteers in the study. All authors revised the manuscript critically for important intellectual content, read and approved the final manuscript.

## Supplementary Material

Additional file 1 Animation showing the pneumatic driven compression device and the gelatine phantom. Click here for file

Additional file 2 Appendix with detailed method description. Click here for file

Additional file 3 Animation showing the complete work flow from loading images, delineation, strain calculation and manual corrections. Click here for file

Additional file 4 Animation showing the results of the optical tracking including the position of the three ROI's used for comparison between strain from optical tracking and from velocity encoded CMR imaging. Click here for file

Additional file 5 Animation of the phantom showing longitudinal strain derived from the velocity encoded CMR images. Click here for file

Additional file 6 Animation showing longitudinal strain in a healthy volunteer in a three chamber image. Click here for file

Additional file 7 Animation showing longitudinal strain in a two chamber image in a patient with an inferior infarct. Click here for file
