# Supplementary material for: Longitudinal strain from velocity encoded cardiovascular magnetic resonance: a validation study
Source: J Cardiovasc Magn Reson. 2013 Jan 23;15(1):15. doi: 10.1186/1532-429X-15-15 (PMC3562217; doi:10.1186/1532-429X-15-15)
Supplement: Additional file 2 — Appendix with detailed method description. [file 1532-429X-15-15-S2.pdf]

Appendix

This appendix describes the motion tracking and strain calculation algorithm in detail. Starting from velocity encoded data the myocardium is tracked throughout the cardiac cycle.

The myocardium is represented as a spatiotemporal finite-element mesh that undergoes a non-rigid cyclic motion.

In an ideal case without noise the displacement of mesh-point could be calculated as follows:

$$\dot{u}(x,\ t) = \hat{v}(x + u(x,\ t),\ t) \tag{A1}$$

where $u(x,\ t)$ is the displacement of a node over time *t*, $\hat{v}(x)$ is the measured velocity, and $u(x,\ 0) = 0$. The goal is now to construct a robust method of solving (A1) also in the presence of noise and artefacts. To do this we solve an optimization problem where we minimize the difference with the measured velocities and the time derivative of the tracked nodes as:

$$\min \frac{1}{2} \int_0^T \int (\dot{u} - \hat{v}(x+u))^2 \tag{A2}$$

where $u(x,\ t)$ is the displacement of a node over time *t*, $\hat{v}(x)$ is the measured velocity.

Instead of solving Equation A2, directly for the deformation *u*, we instead represent the deformation on the boundary with local basis functions. This will introduce spatial regularity and smoothness. The deformation in the interior of the myocardium is based on the deformation at the boundary and assuming a material model. Furthermore, the whole computation process needs to be able to handle constraints in form of user supplied manual corrections of the deformation when necessary for an accurate tracking.

A flowchart of the entire algorithm is given in Figure A1.

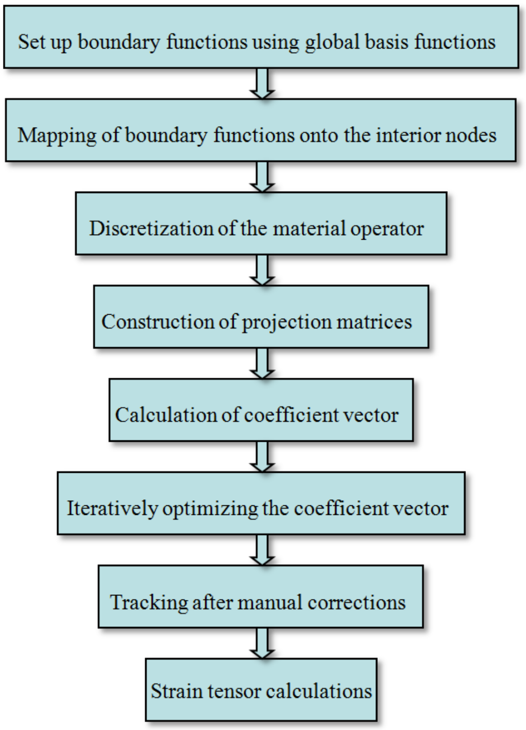

Figure A1. Flowchart of the complete motion tracking and strain calculation algorithm

The optimization problem is solved iteratively. The geometry of myocardium is approximated by manual delineation of myocardium boundary on the long axis view in the first time frame. The region is divided into small triangular elements using Delaunay triangulation algorithm. An example of the triangulated mesh is illustrated in Figure A2. The calculated deformation provides the mesh configuration at the subsequent time frames. In the following algorithm description we will differentiate between boundary nodes, which are finite element nodes that reside on the boundary of the myocardium. Internal nodes are finite element nodes that do not lie on the boundary of the myocardium.

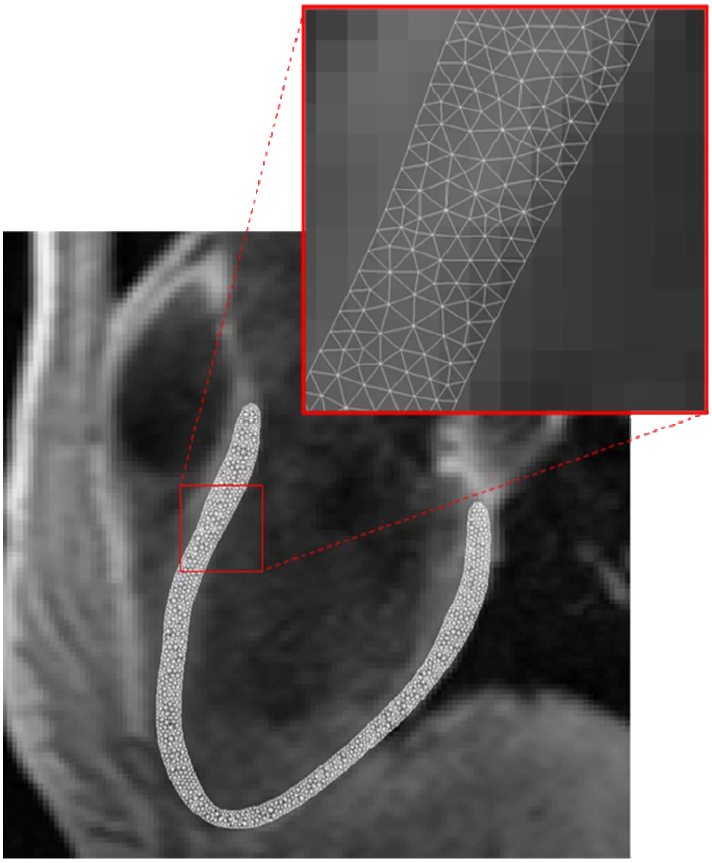

Figure A2. The image shows the finite element mesh in the first timeframe of a typical healthy normal. The red box indicates location of a zoom to show typical mesh density.

Step 1: Set up boundary function

The deformation of each boundary node is calculated from a set of local basis functions. Seven local basis functions are used and their centrums are located equally distributed along the center line of the myocardium. For each local basis function the following four modes of motion are allowed; radial expansion, shear, and outward motion, longitudinal contraction (including passive motion). These four modes of motion have been chosen to be physiologically plausible. The longitudinal motion deserves a special comment. Consider a case where the there is only contraction in a midventricular section, no contraction either basally or apically. Then we would expect that the basal portion of the ventricle is passively dragged towards the apex (no

longitudinal strain). This is the purpose of including the passive motion in longitudinal contraction mode. In order to describe the motion modes algebraically we define α_i is the local angle of the node i, d_i as the distance of the node i to the centre line (defined as positive on the endocardial side of the centre line, and negative on the epicardial centre line), a_i the coordinate of the node i along the arc length, where the arc length is normalized to -1..1, a_j is the coordinate o the centre of the local basis function j along the arc length, w_{ij} as a weighting function for node i with respect to local basis j, p_{ij} is a weighting function that includes passive motion of longitudinal contraction for node i and basis function j. The index i goes from 1 ... number of boundary points, and index j goes from 1..7. The weighting function is calculated as:

$$w_{ij} = \exp(-(a_i - a_j)^2/\sigma) \quad \text{(A3)}$$

where $\sigma = 0.05$. The weighting function that includes passive motion of longitudinal contraction was defined as:

$$p_{ij} = \mathrm{sign}(a_j)\ \mathrm{iexp}(-(a_i - a_j)^2/\sigma) \quad \text{(A4)}$$

where sign(a_j) is the sign function defined as -1 for a_j<0, and 1 for a_j>0, iexp is the integral of the exponential function.

Radial motion for node i and basis function j was defined as:

$$\begin{aligned} r_{xij} &= -d_i\, w_{ij} \sin(\alpha_i) \text{ where } d_i > 0\text{, zero otherwise} \\ r_{yij} &= -d_i\, w_{ij} \cos(\alpha_i) \text{ where } d_i > 0\text{, zero otherwise} \end{aligned} \quad \text{(A5)}$$

Shear motion for node i and basis function j was defined as:

$$\begin{aligned} s_{xij} &= -d_i\, w_{ij} \cos(\alpha_i) \\ s_{yij} &= d_i\, w_{ij} \sin(\alpha_i) \end{aligned} \quad \text{(A6)}$$

Outward motion for node *i* and basis function *j* was defined as:

$$o_{xij} = w_{ij} \sin(\alpha_i)$$
$$o_{yij} = w_{ij} \cos(\alpha_i) \qquad \text{(A7)}$$

Finally the longitudinal motion including passive motion was defined as:

$$l_{xij} = -p_{ij} \cos(\alpha_i)$$
$$l_{yij} = p_{ij} \sin(\alpha_i) \qquad \text{(A8)}$$

The basis function are formulated in a matrix notion. This matrix is denoted as *P* and is a sparse matrix with 4 *j* columns and one row for each of the boundary node *i*.

$$P = \begin{bmatrix} r_{x11} & s_{x11} & o_{x11} & l_{x11} & \cdots & r_{x1j} & s_{x1j} & o_{x1j} & l_{x1j} \\ \cdots \\ r_{xi1} & s_{xi1} & o_{xi1} & l_{xi1} & \cdots & r_{xij} & s_{xij} & o_{xij} & l_{xij} \\ \cdots \\ r_{y11} & s_{y11} & o_{y11} & l_{y11} & \cdots & r_{y1j} & s_{y1j} & o_{y1j} & l_{y1j} \\ \cdots \\ r_{yi1} & s_{yi1} & o_{yi1} & l_{yi1} & \cdots & r_{yij} & s_{yij} & o_{yij} & l_{yij} \end{bmatrix} \qquad \text{(A9)}$$

Step 2: Mapping of boundary functions onto the interior nodes

As previously mentioned we are only explicitly calculating the deformation of the boundary nodes. The displacement of the interior nodes is introduced in the calculation by using a matrix *G*, which is constructed by adjoining zeroes to matrix *P* (from Step 1). The process is illustrated in the following example:

Suppose the index of boundary nodes are 1,3,5,6,7,9,10,11 and index of interior nodes are 2, 4, and 8. *G* is constructed such that:

Rows 2, 4, and 8 are zero rows.

- Rows 1, 3,5,6,7,9,10, and 11 are unit rows.

$$G = \begin{bmatrix} 1 & 0 & 0 & 0 & 0 & 0 & 0 & 0 \\ 0 & 0 & 0 & 0 & 0 & 0 & 0 & 0 \\ 0 & 1 & 0 & 0 & 0 & 0 & 0 & 0 \\ 0 & 0 & 0 & 0 & 0 & 0 & 0 & 0 \\ 0 & 0 & 1 & 0 & 0 & 0 & 0 & 0 \\ . & . & . & . & . & . & . & . \\ . & . & . & . & . & . & . & . \end{bmatrix} \tag{A10}$$

The matrix G will be used in step 4.

Step 3: Discretization of the material operator

The Cauchy-Navier operator $\mathcal{N}$ is used to ensure spatial smoothness and spatial continuity of the deformation. Since we are tracking a periodically moving and deforming object, we consider a finite element mesh whose node points are attached to a set of material points of the underlying myocardium, such that the mesh moves and deforms along with the material region. The use of the Cauchy-Navier operator can be seen as way of adding linear material properties in the tracking formulation. The material operator $\mathcal{N}$ is discretized by finite element operators. This is formulated in matrix notation as:

$$\mathcal{N} = \begin{bmatrix} (1+k)\frac{\partial}{\partial x^2} + \frac{\partial}{\partial y^2} & k\frac{\partial}{\partial y \partial x} \\ k\frac{\partial}{\partial x \partial y} & \frac{\partial}{x^2} + (1+k)\frac{\partial}{\partial y^2} \end{bmatrix} \tag{A11}$$

where k are $1/(1\text{-}2v)$ where v is a material property constant and was set to 0.45, $\frac{\partial}{\partial x^2} = (D_x)^T M (D_x)$, $\frac{\partial}{\partial y \partial x} = (D_y)^T M (D_x)$, D_x and are derivative matrices constructed from the derivative values at each node of all the triangular elements with respect to x and y respectively, and M is a diagonal matrix with diagonal entries as the areas of the triangular elements. $\mathcal{N}$ is defined for both boundary nodes and interior nodes.

Step 4: Construction of projection matrices

The displacements of all the nodes (both boundary and interior) are calculated as a matrix multiplication.

$$u = Ac \tag{A13}$$

where c is a coefficient vector of basis functions. A is a mapping matrix for all nodes, and is constructed as

$$A = \mathcal{N}^{-1}GP \tag{A14}$$

where $\mathcal{N}$ is the Cauchy-Navier operator, P is the basis function matrix, and G is the mapping matrix onto the interior nodes. $\mathcal{N}$, P and G are defined in the previous steps. When taking the temporal derivative of Equation A13 twice we get the following

$$\frac{\partial^2 u}{\partial t^2} = A\frac{\partial^2 c}{\partial t^2} \tag{A15}$$

This can be expressed in matrix notation as

$$\frac{\partial^2 u}{\partial t^2} = AHc \tag{A16}$$

where H is a temporal second derivative operator written as

$$H = \begin{bmatrix} -2 & 1 & 0 & 0 & 0 & 0 & 0 & 0 \\ 1 & -2 & 1 & 0 & 0 & 0 & 0 & 0 \\ 0 & 1 & -2 & 1 & 0 & 0 & 0 & 0 \\ 0 & 0 & 1 & -2 & 1 & 0 & 0 & 0 \\ 0 & 0 & 0 & 1 & -2 & 1 & 0 & 0 \\ . & . & . & . & 1 & -2 & 1 & . \\ . & . & . & . & . & 1 & -2 & 1 \\ . & . & . & . & . & . & 1 & -2 \end{bmatrix} \tag{A17}$$

The Equation A16 can be re-arranged into

$$
\begin{aligned}
&AHc = \frac{\partial^2 u}{\partial t^2} \\
&Hc = K\frac{\partial^2 u}{\partial t^2} \\
&where,\ K = (A^T A)^{-1} A^T
\end{aligned}
\tag{A18}
$$

where $K = (A^T A)^{-1} A^T$ is pseudo inverse of A.

Step 5: Calculation of coefficient vector

From Equation A12 the coefficient vector c is obtained as follows

$$
\begin{aligned}
&Hc = J \\
&where, J = K\frac{\partial^2 u}{\partial t^2} \\
&\Rightarrow c = H^{-1} J
\end{aligned}
\tag{A19}
$$

where $\frac{\partial^2 u}{\partial t^2}$ (the temporal second derivative of displacement) is calculated using forward differentiation of the measure velocity data.

Step 6: Iteratively optimizing the coefficient vector

The coefficient vector c is calculated according to Equation A19. The displacements of all nodes are calculated according to Equation A13. The new displacements are needed since we interpolate the measured velocity field at the displaced node coordinates (left hand side in Equation A16). This iterative process for calculating the coefficient vector is repeated 10 times.

Step 7: Tracking after manual corrections

The user interface for manual interaction allows the user to pre-specify the displacement of specific boundary node(s) at specific time frame(s). The nearest boundary node to each manual correction is stored. To maintain local dependency of manual corrections, the boundary functions are reset by including local first order polynomial basis into the matrix P (for details see Step 8) which give rise to matrix P_{new}. Using P_{new}, matrices A and K are recalculated as given in Equation A20.

$$A_{new} = N^{-1} G P_{new}$$
$$K_{new} = (A_{new}^{\mathrm{T}} A_{new})^{-1} A_{new}^{\mathrm{T}} \tag{A20}$$

Each manual correction introduces a constraint ($u = Ac$) into the system of linear equations ($Hc = J$) of coefficients c. We are forming a new matrices H_{new} and J_{new}, where we add one displacement constraint one for each manual correction. Now we can again iteratively solve for the coefficient vector c, but now instead of using Equation A19, we use the following equation

$$\mathrm{c} = (H_{new})^{-1} J_{new} \tag{A21}$$

This is repeated 10 times.

Step 9: Strain tensor calculations

As a last step the strain tensor is calculated. First the displacements of all nodes are calculated according to Equation A13. If u_x and u_y are the displacement components in x and y directions, then 2 dimensional strain components ε_x, ε_y and γ_{xy} on the faces of a strain element aligned with the xy coordinate system are obtained as follows:

$$\varepsilon_x = \frac{\partial u_x}{\partial x},\ \varepsilon_y = \frac{\partial u_y}{\partial y},\ \gamma_{xy} = \frac{\partial u_x}{\partial y} + \frac{\partial u_y}{\partial x} \tag{A22}$$

If the motion of myocardium is described in lateral (x') and axial (y') directions, then the strain components $\varepsilon_{x'}$ (longitudinal strain), $\varepsilon_{y'}$ (radial strain) and $\gamma_{x'y'}$ (shear strain) with respect to an element aligned with the $x'y'$ coordinate system, rotated by angle θ from xy are:

$$\begin{aligned}
\varepsilon_{x'} &= \varepsilon_x \cos^2\theta + \varepsilon_y \sin^2\theta + 2\gamma_{xy} \sin\theta\cos\theta \\
\varepsilon_{y'} &= \varepsilon_x \sin^2\theta + \varepsilon_y \cos^2\theta - 2\gamma_{xy} \sin\theta\cos\theta \\
\gamma_{x'y'} &= -(\varepsilon_x - \varepsilon_y)\sin 2\theta + \gamma_{xy}\cos 2\theta
\end{aligned} \tag{A23}$$
